# Supplementary material for: Genomic and fitness consequences of a near-extinction event in the northern elephant seal
Source: Nat Ecol Evol. 2024 Sep 27;8(12):2309–24. doi: 10.1038/s41559-024-02533-2 (PMC11618080; doi:10.1038/s41559-024-02533-2)
Supplement: Supplementary file 1 — Supplementary Results and Discussion, Tables 1–4 and References. [file 41559_2024_2533_MOESM1_ESM.pdf]

nature ecology & evolution

Supplementary information https://doi.org/10.1038/s41559-024-02533-2

Genomic and fitness consequences of a near-extinction event in the northern elephant seal

In the format provided by the authors and unedited

Supplementary information for:

Genomic and fitness consequences of a near-extinction event in the northern elephant seal

Joseph I. Hoffman, David L. J. Vendrami, Kosmas Hench, Rebecca Chen, Martin A. Stoffel, Marty Kardos, William Amos, Joern Kalinowski, Daniel Rickert, Karl Köhrer, Thorsten Wachtmeister, Mike E. Goebel, Carolina A. Bonin, Frances M.D. Gulland & Kanchon K. Dasmahapatra

Correspondence: Joseph I. Hoffman, joseph.hoffman@uni-bielefeld.de, Department of Evolutionary Population Genetics, Faculty of Biology, Bielefeld University, Bielefeld, Germany.

Supplementary results and discussion **Pages 2–4**

Supplementary tables **Pages 5–9**

References **Pages 10–11**

Supplementary results and discussion

Inbreeding depression

Following Acevedo-Whitehouse et al.[1], we defined trauma as a "control" category and implemented Bayesian multinomial GLMMs comparing each category with trauma as a reference. The 95% CIs of the posterior distributions of the standardized beta coefficients of sMLH all overlapped zero (Extended Data Fig. 2a, Supplementary Table 3) suggesting that levels of inbreeding do not differ between any of the categories and animals that died from trauma. We additionally implemented binomial GLMMs comparing the trauma category with all of the other categories combined. The 95% CIs of the posterior distributions of the standardized beta coefficients of sMLH again overlapped zero (Extended Data Fig. 2b, microsatellites: median $\beta = 0.26$, 95% CI = -0.23–0.82; SNPs: median $\beta = 0.36$, 95% CI = -0.41–1.25), suggesting that there is no difference in inbreeding between sick animals and otherwise healthy animals that died from trauma.

Our study design provides us with unusually detailed veterinary pathology data because the animals originated from a marine mammal recovery centre. The samples were assembled over several years and originated from a wide geographic area. Consequently, we believe our sample is representative of the wider population, rather than representing, for example, animals from a single breeding colony. However, our sample is enriched for sick animals, raising the question of whether this creates a sampling bias. We believe not, for two reasons. First, the trauma group should be random with respect to inbreeding, because there is no reason to expect inbreeding depression for accidental injuries such as boat strikes. Second, a genetic bias could potentially arise if animals either carrying or lacking specific alleles at a given locus differed in their disease susceptibility. However, such a bias would not be genome-wide, but instead would apply only to that particular locus and strongly linked regions, leaving the rest of the genome unaffected.

Demographic reconstruction

As a confirmatory step, we repeated the demographic analyses using whole genome sequencing (WGS) data from a representative subset of 20 individuals as described in the Methods. The six-generation bottleneck model received the greatest support (Supplementary Table 4). Regardless of the length of the inferred bottleneck, there was a high degree of concordance between the parameter estimates obtained from the bottleneck models for the RAD sequencing and WGS datasets (Extended Data Fig. 5, Supplementary Table 4). The

main differences were that the best WGS-based estimate of $N_{ePOSTBOT}$ was larger (point estimate = 6,248, 95% CI = 3,708–16,824) while the corresponding N_{eBOT} estimate was smaller (point estimate = two, 95% CI = 1–3). Due to the overall similarity of the results and the fact that the greater sample size of individuals in the RAD sequencing dataset results in a larger number of frequency classes being resolved in the SFS, we focused subsequent analyses on the results of the demographic reconstruction based on the RAD sequencing data, although we also explored the sensitivity of our results to different datasets and assumptions.

Genetic load simulations

To evaluate the robustness of our results, we repeated the WF simulations while relaxing our assumptions as well as using different datasets. First, we allowed deleterious mutations to occur only in exons while keeping $U \sim 1.2$, but this did not significantly affect the results (Extended Data Fig. 7a–d). Second, we incorporated uncertainty in the demographic estimates by re-running the simulations using the bootstrapped N_e estimates from the demographic model. We observed more variation in the total and inbreeding load before and during the bottleneck, reflecting uncertainty in the $N_{ePREBOT}$ estimates. However, the results for the post-bottleneck population converged on similar values to those obtained in the original simulations (Extended Data Fig. 7e–h) suggesting that N_{eBOT} is the main parameter affecting the various load components of the post-bottleneck population. Finally, we repeated the simulations using point N_e estimates from the demographic model based on the WGS data. Again, the results were similar (Extended Data Fig. 7i–l), although the post-bottleneck population had a marginally lower inbreeding load and a marginally higher drift load, probably due to stronger genetic drift resulting from N_{eBOT} being smaller. Taken together, these findings suggest that our inferences based on the WF models are reasonably robust to the underlying assumptions and datasets used for modelling.

Genomic inbreeding and individual genomic mutation loads

Average individual genome-wide heterozygosity for the contemporary northern elephant seal population (0.00018) was nearly identical to that estimated by Hoelzel et al.[2] (0.000176) and average individual genome-wide heterozygosity for the southern elephant seal (0.00149) was nearly identical to that estimated for the pre-bottleneck northern elephant seals sampled by Hoelzel et al.[2] (0.00142). Assuming that the latter estimate is an accurate reflection of the average pre-bottleneck heterozygosity of the northern elephant seal, the harmonic mean N_e since the onset of the bottleneck can be estimated by solving:

$$f_t = 1 - \left(1 - \left(\frac{1}{2N_e}\right)\right)^t$$

for N_e after setting t (the number of generations since the onset of intensive harvesting) to 23 and f_t (the proportional reduction in average heterozygosity in the post- versus pre-bottleneck population) to 0.00142 – 0.00018 / 0.00142 = 0.87. This yields an estimated harmonic mean N_e of 5.83 for the northern elephant seal over the 23 generations since the onset of the bottleneck, further supporting the conclusion of extremely small N_e during the bottleneck.

Mapping the deleterious mutations to the northern elephant seal reference genome, we found that the inbreeding, segregating and drift loads were broadly distributed across the genomes of both species (Extended Data Fig. 9). The number of mutations per gene ranged between one and 16, with the mean being 1.05 ± 0.27 SD for the northern elephant seal and 1.15 ± 0.64 SD for the southern elephant seal (a table describing the load per gene is available via figshare). In comparison to the southern elephant seal, the northern elephant seal had fewer genes carrying mutations contributing to the inbreeding load (100 versus 896) and the segregating load (60 versus 360) but more genes carrying mutations contributing to the drift load (192 versus 53).

Broadly speaking, our findings are consistent with previous studies of other mammalian species using similar methodologies. For example, small population sizes, geographical isolation and population declines have been associated with reduced burdens of deleterious mutations in Iberian lynx [3], Indian tigers[4] killer whales[5] and vaquita[6], while in island foxes, long-term small N_e appears to be associated with a reduced burden of strongly deleterious alleles and a low prevalence of congenital defects that are commonly associated with inbreeding[7]. However, in Grauer's gorillas, an 80% population decline over the past two decades resulted in many loss of function and missense mutations drifting to higher frequencies[8], while in Alpine ibex, the purging of highly deleterious mutations from strongly bottlenecked populations was accompanied by the accumulation of mildly deleterious mutations[9]. These different outcomes, together with the results of our own study, emphasise the importance of understanding how multiple factors including population-specific demographic histories, life-history variation and chance events[10] shape genomic landscapes of deleterious variation across species.

Supplementary tables

Supplementary Table 1. Details of the 22 microsatellite loci used to genotype 219 northern elephant seal individuals. "Multiplex" denotes the PCR mastermix into which each locus was multiplexed and "T_a" denotes the annealing temperature used. H_e: expected heterozygosity; H_e: observed heterozygosity. Hardy-Weinberg equilibrium (HWE) *p*-values (two-sided tests) are shown. None of these were statistically significant after table-wide correction for the false discovery rate.

Locus	Isolated from species	Literature source	Genbank accession number	Multiplex	T_a (°C)	Number of alleles	H_e	H_o	HWE *p*-value
Hg3.6	Grey seal, *Halichoerus grypus*	Allen *et al.*[11]	G02090	1	50	2	0.197	0.185	0.315
Pv9	Harbour seal, *Phoca vitulina*	Allen *et al.*[11]	G02096	1	50	2	0.456	0.440	0.661
PvcA	Harbour seal, *Phoca vitulina*	Coltman *et al.*[12]	L40983	2	50	3	0.657	0.688	0.739
M11a	Southern elephant seal, *Mirounga leonina*	Gemmell *et al.*[13]	–	4	60	2	0.308	0.304	0.827
BG	Southern elephant seal, *Mirounga leonina*	Gemmell *et al.*[13]	–	4	60	2	0.217	0.247	0.029
Lw20	Weddell seal, *Leptonychotes weddellii*	Gelatt *et al.*[14]	AF140595.1	1	50	3	0.660	0.673	0.852
ZcCgDh1.8	California sea lion, *Zalophus californianus*	Hernandez-Velazquez *et al.*[15]	AY676475	4	60	2	0.280	0.300	0.337
ZcCgDh3.6	California sea lion, *Zalophus californianus*	Hernandez-Velazquez *et al.*[15]	AY676476	4	60	2	0.438	0.502	0.033
ZcCgDh4.7	California sea lion, *Zalophus californianus*	Hernandez-Velazquez *et al.*[15]	AY676478	3	50	3	0.511	0.478	0.659
ZcwA12	Galápagos sea lion, *Zalophus californianus wollebaeki*	Hoffman *et al.*[16]	DQ836320	3	50	2	0.477	0.430	0.161
ZcwC01	Galápagos sea lion, *Zalophus californianus wollebaeki*	Hoffman *et al.*[16]	DQ836323	2	50	2	0.365	0.380	0.712

ZcwE04	Galápagos sea lion, *Zalophus californianus wollebaeki*	Hoffman *et al.*[16]	DQ836324	3	50	2	0.180	0.190	0.702
ZcwF07	Galápagos sea lion, *Zalophus californianus wollebaeki*	Hoffman *et al.*[16]	DQ836326	2	50	2	0.449	0.533	0.016
ZcwG04	Galápagos sea lion, *Zalophus californianus wollebaeki*	Hoffman *et al.*[16]	DQ836328	1	50	3	0.518	0.534	0.934
71HDZ441	Steller's sea lion, *Eumetopias jubatus*	Huebinger *et al.*[17]	DQ777849	1	50	2	0.494	0.475	0.584
Hl-18	Leopard seal, *Hydrurga leptonyx*	Davis *et al.*[18]	AF140585	1	50	3	0.635	0.649	0.917
Mang01	Northern elephant seal, *Mirounga angustirostris*	Sanvito *et al.*[19]	JQ714261.1	1	50	3	0.467	0.493	0.358
Mang06	Northern elephant seal, *Mirounga angustirostris*	Sanvito *et al.*[19]	JQ714265.1	3	50	2	0.361	0.363	1.000
Mang27	Northern elephant seal, *Mirounga angustirostris*	Sanvito *et al.*[19]	JQ714272.1	1	50	2	0.178	0.188	0.703
Mang35	Northern elephant seal, *Mirounga angustirostris*	Sanvito *et al.*[19]	JQ714275.1	2	50	3	0.613	0.596	0.307
Mang36	Northern elephant seal, *Mirounga angustirostris*	Sanvito *et al.*[19]	JQ714276.1	4	60	2	0.197	0.185	0.315
Mang44	Northern elephant seal, *Mirounga angustirostris*	Sanvito *et al.*[19]	JQ714281.1	1	50	2	0.456	0.439	0.661

Supplementary Table 2. Point estimates and 95% CIs of the standardized beta coefficients of sMLH on body mass and blubber thickness obtained from Bayesian linear mixed models.

Markers	Trait	Median	95% CI
22 microsatel-lites	Body mass (kg)	-0.09	[-1.21, 1.05]
	Blubber thickness (cm)	0.04	[-0.05, 0.12]
15,051 SNPs	Body mass (kg)	0.38	[-0.84, 1.52]
	Blubber thickness (cm)	0.02	[-0.14, 0.20]

Supplementary Table 3. Point estimates and 95% CIs of the standardized beta coefficients of sMLH on the most likely cause of death. On the left are shown the results of separate Bayesian generalized linear mixed models (GLMMs) for each category, using a binomial response variable with 1 indicating that the respective disease or condition was the most likely cause of death of a given individual. On the right are shown the results of a single Bayesian multinomial GLMM, where the sMLH of each category was compared to trauma as a reference.

Binomial GLMMs				Multinomial GLMM			
**Markers**	**Cause of death**	**Median**	**95% CI**	**Markers**	**Cause of death**	**Median**	**95% CI**
22 microsatellites	Helminth infection	0.03	[-0.35, 0.43]	22 microsatellites	Helminth infection	0.13	[-0.39, 0.47]
	Bacterial infection	0.07	[-0.34, 0.49]		Bacterial infection	0.11	[-0.43, 0.69]
	Protozoal infection	-0.29	[-1.40, 0.72]		Protozoal infection	-0.55	[-1.74, 0.48]
	Trauma	-0.27	[-0.82, 0.26]		Trauma	–	–
	Malnutrition	0.03	[-0.25, 0.36]		Malnutrition	0.11	[-0.38, 0.61]
	Congenital defect	0.09	[-0.41, 0.57]		Congenital defect	0.14	[-0.51, 0.75]
15,051 SNPs	Helminth infection	-0.06	[-0.75, 0.57]	15,051 SNPs	Helminth infection	-0.21	[-0.98, 0.56]
	Bacterial infection	-0.15	[-0.89, 0.47]		Bacterial infection	-0.29	[-1.19, 0.54]
	Protozoal infection	0.00	[-1.21, 1.10]		Protozoal infection	-0.44	[-1.99, 0.75]
	Trauma	-0.39	[-1.27, 0.34]		Trauma	–	–
	Malnutrition	0.30	[-0.54, 1.04]		Malnutrition	0.25	[-0.64, 1.13]
	Congenital defect	0.28	[-0.67, 1.14]		Congenital defect	0.37	[-0.76, 1.40]

Supplementary Table 4. Relative likelihoods of alternative demographic models together with AIC values and parameter estimates for the RAD sequencing (RAD) and whole genome sequencing (WGS) datasets. Three alternative demographic models were evaluated for each dataset, the first including a bottleneck lasting for six generations (bot06), the second including a bottleneck lasting for ten generations (bot10) and a "null" model that did not include a bottleneck. The point estimates were obtained from the model with the best likelihood among 100 independent runs for each model. Shown in parentheses are the median and 95% confidence interval calculated from 100 datasets bootstrapped over individuals. N_{eLGM}: effective population size during the last glacial maximum; $N_{ePREBOT}$: effective population size before sealing; N_{eBOT}: effective population size during the bottleneck; $N_{ePOSTBOT}$: effective population size in the current day; T_{se}: time of the end of the LGM in generations ago.

**Method**	**Model**	**k**	**AIC**	**Estimated likelihood**	**Observed likelihood**	N_{eLGM}	$N_{ePREBOT}$	N_{eBOT}	$N_{ePOSTBOT}$	T_{se}
RAD	bot06	5	78654	-17077	-16686	267 (812, [227, 1722])	12856 (5904, [2828, 20275])	6 (6, [5, 7.5])	2624 (2730, [2506, 2773])	1222 (878, [351, 1570])
RAD	bot10	5	78878	-17126	-16686	488 (1037, [422, 1990])	5722 (3796, [2746, 13815])	11 (12, [10, 14])	2590 (2731, [2511, 2838])	1145 (785, [304, 1346])
RAD	null	3	81302	-17654	-16686	2186 (2298, [2132, 2424])	–	–	2601 (2683, [2514, 2756])	101 (101, [101, 102])
WGS	bot06	5	6220715	-1350809	-1349808	706 (724, [330, 1583])	11836 (7091, [3297, 14763])	2 (2, [1, 3])	6248 (10320, [3708, 16824])	548 (558, [209, 956])
WGS	bot10	5	6220717	-1350809	-1349808	790 (747, [343, 1725])	5602 (6778, [3319, 14732])	4 (2, [2, 5])	13590 (10477, [3884, 16624])	677 (576, [237, 944])
WGS	null	3	6264779	-1360378	-1349808	2822 (2868, [2753, 3016])	-	-	2770 (2819, [2693, 2962])	133 (143, [119, 174])

References

1 Acevedo-Whitehouse, K., Gulland, F., Greig, D. & Amos, W. Inbreeding: Disease susceptibility in California sea lions. *Nature* **422**, 35, doi:10.1038/422035a (2003).

2 Hoelzel, A. R. *et al.* Genomics of post-bottleneck recovery in the northern elephant seal. *Nature Ecology and Evolution* **8**, 686–694, doi:10.1038/s41559-024-02337-4 (2024).

3 Kleinman-Ruiz, D. *et al.* Purging of deleterious burden in the endangered Iberian lynx. *Proceedings of the National Academy of Sciences* **119**, e2110614119, doi:10.1073/pnas.2110614119 (2022).

4 Khan, A. *et al.* Genomic evidence for inbreeding depression and purging of deleterious genetic variation in Indian tigers. *Proceedings of the National Academy of Sciences* **118**, e2023018118, doi:10.1073/pnas.2023018118 (2021).

5 Kardos, M. *et al.* Inbreeding depression explains killer whale population dynamics. *Nature Ecology and Evolution* **7**, 675–686, doi:10.1038/s41559-023-01995-0 (2023).

6 Robinson, J. A. *et al.* The critically endangered vaquita is not doomed to extinction by inbreeding depression. *Science* **376**, 635–639 (2022).

7 Robinson, J. A., Brown, C., Kim, B. Y., Lohmueller, K. E. & Wayne, R. K. Purging of strongly deleterious mutations explains long-term persistence and absence of inbreeding depression in island foxes. *Current Biology* **28**, 3487–3494 (2018).

8 Van Der Valk, T., Díez-Del-Molino, D., Marques-Bonet, T., Guschanski, K. & Dalén, L. Historical genomes reveal the genomic consequences of recent population decline in eastern gorillas. *Current Biology* **29**, 165–170, doi:10.2139/ssrn.3254908 (2019).

9 Grossen, C., Guillaume, F., Keller, L. F. & Croll, D. Purging of highly deleterious mutations through severe bottlenecks in Alpine ibex. *Nature Communications* **11**, 1001, doi:10.1038/s41467-020-14803-1 (2020).

10 Bouzat, J. L. Conservation genetics of population bottlenecks: the role of chance, selection and history. *Conservation Genetics* **11**, 463–478, doi:10.1007/s10592-010-0049-0 (2010).

11 Allen, P. J., Amos, W., Pomeroy, P. P. & Twiss, S. D. Microsatellite variation in grey seals (*Halichoerus grypus*) shows evidence of genetic differentiation between two British breeding colonies. *Molecular Ecology* **4**, 653–662, doi:10.1111/j.1365-294x.1995.tb00266.x (1995).

12 Coltman, D. W., Bowen, W. D. & Wright, J. M. PCR primers for harbour seal (*Phoca vitulina concolour*) microsatellites amplify polymorphic loci in other pinniped species. *Molecular Ecology* **5**, 161–163, doi:10.1111/j.1365-294x.1996.tb00303.x (1996).

13 Gemmell, N. J., Allen, P. J., Goodman, S. J. & Reed, J. Z. Interspecific microsatellite markers for the study of pinniped populations. *Molecular Ecology* **6**, 661–666, doi:10.1046/j.1365-294x.1997.00235.x (1997).

14 Gelatt, T. S., Davis, C. S., Siniff, D. B. & Strobeck, C. Molecular evidence for twinning in Weddell seals (*Leptonychotes weddellii*). *Journal of Mammalogy* **82**, 491–499, doi:https://doi.org/10.1644/1545-1542(2001)082<0491:MEFTIW>2.0.CO;2 (2001).

15 Hernandez-Velazquez, F. D. *et al.* New polymorphic microsatellite markers for California sea lions (*Zalophus californianus*). *Molecular Ecology Notes* **5**, 140–142, doi:10.1111/j.1471-8286.2004.00858.x (2005).

16 Hoffman, J. I., Steinfartz, S. & Wolf, J. B. W. Ten novel dinucleotide microsatellite loci cloned from the Galápagos sea lion (*Zalophus californianus wollebaeki*) are polymorphic in other pinniped species. *Moleular Ecology Notes* **7**, 103–105, doi:10.1111/j.1471-8286.2006.01544.x (2007).

17 Huebinger, R. M., Louis Jr, E. E., Gelatt, T., Rea, L. D. & Bickham, J. W. Characterization of eight microsatellite loci in Steller sea lions (*Eumetopias jubatus*). *Molecular Ecology Notes* **7**, 1097–1099, doi:10.1111/j.1471-8286.2007.01790.x (2007).

18 Davis, C. S., Gelatt, T. S., Siniff, D. & Strobeck, C. Dinucleotide microsatellite markers from the Antarctic seals and their use in other pinnipeds. *Molecular Ecology Notes* **2**, 203–208, doi:10.1046/j.1471-8286.2002.00187.x-i2 (2002).

19 Sanvito, S. *et al.* Isolation and cross-species amplification of novel microsatellite loci in a charismatic marine mammal species, the northern elephant seal (*Mirounga angustirostris*). *Conservation Genetics Resources* **5**, 93–96, doi:10.1007/s12686-012-9741-3 (2013).
